# Depression and Its Relationship with Coping Strategies and Illness Perceptions during the COVID-19 Lockdown in Greece: A Cross-Sectional Survey of the Population

**DOI:** 10.1155/2020/3158954

**Published:** 2020-08-26

**Authors:** Petros Skapinakis, Stefanos Bellos, Achilleas Oikonomou, Georgios Dimitriadis, Paschalis Gkikas, Evridiki Perdikari, Venetsanos Mavreas

**Affiliations:** ^1^Department of Psychiatry, University of Ioannina School of Medicine, Ioannina, Greece; ^2^Private Psychiatric Practice, Greece

## Abstract

**Objective:**

The COVID-19 epidemic has shown a more benign course in Greece possibly due to the early lockdown measures. Mental health consequences of the lockdown however are unknown. In addition, illness perceptions and relevant strategies to cope with the stress of the epidemic may have played a role in complying with the restrictions. We conducted a survey of the Greek population with the aim to investigate the prevalence of anxiety and depression during the lockdown, the emotional impact of the epidemic, and the effect of coping strategies and illness perceptions in mental health.

**Methods:**

Adult persons were invited during the peak of the lockdown period through social media. Depressive and anxiety symptoms were assessed with the PHQ-9 and GAD-2 scales, respectively. Coping strategies were assessed with selected items of the Brief COPE questionnaire, while illness beliefs were assessed with items from the revised Illness Perception Questionnaire (IPQ-R).

**Results:**

A total of 3379 individuals took part. A strong emotional impact of the epidemic was more often in women and in those with severe financial difficulties. Levels of depressive and anxiety symptoms were high but similar to past assessments. Participants showed high levels of personal control and used more often positive strategies to cope with the stress of the epidemic. Depressive symptoms were higher in the younger, in students, in those with a stronger emotional impact, in those isolated due to symptoms, and those overexposed to media for COVID-19-related news. Lower levels of depression were seen in those using positive coping strategies and showing high levels of personal and treatment control.

**Conclusions:**

The study shows that certain psychological and social determinants were associated with increased depressive symptoms during the lockdown warranting the development of public health guidelines to mitigate the effects of the epidemic to the mental health of the population.

## 1. Introduction

The COVID-19 pandemic may have important psychological and social effects, but these have not been assessed at the population level [1]. Apart from the fear of a potentially severe disease with no specific treatment, there is also increasing concern that the mental health consequences of the social distancing measures and the lockdown may be comparable or even exceed the burden of physical illness and mortality due to the virus itself [2]. Thus, a more thorough examination of the mechanisms and determinants that are associated with increased psychological symptoms as a result of the COVID-19 pandemic would be useful from a clinical and public health perspective.

Previous research has shown that the psychological impact of health-related stressful events, including chronic diseases or infectious outbreaks, is influenced by the specific beliefs about the illness and the resulting coping behaviors that people tend to use in order to adapt better and mitigate the mental health consequences [3, 4].

“Coping” is the set of psychological responses to perceptions of threat with the aim to prevent or diminish threat, harm, and loss or to reduce associated distress [5]. Meta-analyses indicate that engagement with a set of “functional” coping strategies is associated with better physical and mental health in individuals facing a range of stressors, such as traumatic events (social stress, bullying) or health-related conditions (HIV, cancer, and diabetes) [6–11]. In addition, outbreaks of viral infections are considered among the conditions that coping strategies have been found to play an important role on emotional responses and the development of psychological problems [4, 12–14].

Choice of coping behaviors may be influenced by specific illness perceptions defined as the set of beliefs that the individuals develop for a health condition [15]. Some studies have assessed Illness perceptions for a hypothetical physical health problem [16–18] or chronic infectious disease [19–21] and found correlations between illness perceptions and attitudes or intentions towards the adoption of preventive behaviors or use of health services.

Illness perceptions and coping behaviors related to the COVID-19 pandemic could be important as they may be associated with the increased psychological burden of depression and anxiety that has been reported mainly in health professionals [22]. In addition, specific perceptions and coping strategies may lead to more adaptive health behaviors and rates of compliance with official recommendations, as it was the case in previous viral outbreaks [23, 24]. Previous small surveys have studied the association between the perceptions about a viral infection and the psychological responses to epidemic outbreaks, showing that perceptions about viral infections play an important role [25, 26].

In Greece, the COVID-19 epidemic has shown a more benign trajectory that has been portrayed in the international news media as the “uniqueness of the Greek case,” at least compared with mortality rates of neighboring Italy or other Mediterranean countries such as France or Spain. Restrictions were imposed early, and compliance to the lockdown measures was universal and successful with little opposition from the public. It is important to study any potential mental health consequences of these restrictions. In addition, an investigation of the main coping behaviors of the Greek public and the prevalent perceptions of the illness and the epidemic in general may offer useful suggestions for public health officials and the better implementation of preventive measures elsewhere. Therefore, we undertook the current study with the aim to assess the mental health status of the Greek population during the peak of the COVID-19 lockdown and its association with the main coping behaviors used and the prevalent illness beliefs about the epidemic.

## 2. Methods

### 2.1. Description of the Dataset

We conducted an electronic web survey using Google Forms. Participants were recruited through social media, mainly Facebook. All adults (18 or more) were eligible to participate with no other restrictions other than the language barrier. In order to increase the sample size and the representativeness of the sample, we used both passive and active convenience snowball sampling [27]. We posted the invitation to the study on Facebook, and we encouraged others to share the post (passive snowballing). In addition, we actively sought other health professionals, journalists, and other individuals with strong social media presence to share our invitation. In order to increase the participation of men, younger, and elderly individuals as well as individuals living in both major and small cities, we also requested from individuals with strong social media presence in these subgroups to share the links to our survey. We repeated the procedure on the third day to increase the sample size. We posted the first link to our survey at 9 am on the 8^th^ of April, and we closed the survey at 19.00 on the 12^th^ of April. The percentage of participants recruited at each day was as follows: 39% (8 April), 22% (9 April), 9% (10 April), 24% (11 April), and 6% (12 April). During this period, the lockdown restrictions due to the COVID-19 epidemic were fully implemented in Greece.

The online survey was completely anonymous, and informed consent was obtained from the participants at the beginning of the survey. All participants were free to leave the survey at any point before submitting the results. This research was conducted in accordance with the Helsinki Declaration and the ethical standards of our Institutional Bioethics Committee. A second wave of data collection is also planned in the future, after the easing of the lockdown restrictions and the gradual opening up of the economy.

### 2.2. Assessment of Current Mental Health Status and General Health

Depressive symptoms were assessed with the PHQ-9, the 9-item depression module of the Patient Health Questionnaire [28]. We had previously validated this version in chronic kidney disease patients in Greece [29]. A cut-off of 10 had shown good sensitivity (82%) and specificity (93%) against a diagnosis of depression using a structured diagnostic interview. In the context of the current study in our analyses, we used the total scores on the PHQ-9 (range 0 to 27), and we also report two categories of depressive symptoms using the threshold of 10 for mild (or more) depression and 15 for moderate/severe depression only [28].

Anxiety symptoms were assessed with the first 2 core items of the Generalized Anxiety Disorder scale (GAD-2) [30]. This has shown high sensitivity and specificity to detect GAD in primary care [31]. For the analyses, we used the total scores (range 0 to 6) and two cut-offs at 3 and 4 to denote mild or moderate anxiety, respectively.

Level of general health was assessed with the very well-validated single question regarding global health status [32]. This assesses current general health using a 5-point Likert scale. In the current paper, we derived a binary variable of fair/poor general health versus good (or better) health.

### 2.3. Assessment of Coping Strategies

Coping strategies of the sample during the epidemic were assessed using selected items of the Brief COPE questionnaire [33]. This has been translated and validated in Greek using community samples from Greece and Cyprus [34]. Brief COPE evaluates 14 strategies to cope with stressful events. Each coping strategy is represented by two items. In order to minimize the burden of participants, two of the authors collected one item for each strategy using a consensus procedure. One strategy (self-blame) was excluded as it was considered not applicable to the epidemic. Therefore, we finally included 13 items representing an equal number of coping strategies. Views about coping styles are rated using a four-point Likert scale from “not at all” (coded 1) to “a lot” (coded 4).

### 2.4. Assessment of Illness Perceptions

Illness perceptions related to the COVID-19 epidemic were assessed using the revised “Illness Perception Questionnaire” (IPQ-R) [35]. IPQ-R has been widely used and validated in a range of chronic diseases as well as in healthy participants for hypothetical disease scenarios or genetic predispositions [36, 37]. The original IPQ-R includes 38 cognitive and emotional representations of illness grouped into the following dimensions: timeline of the illness (acute/chronic), timeline (cyclical), personal control, treatment control, illness coherence, emotional representations, and perceived consequences. We adapted the IPQ-R to be used during the COVID-19 epidemic. Two of the authors initially selected 26 of the 38 items. We excluded all items of the illness coherence dimension as these were not applicable to the current epidemic. From the remaining 26 items, we further eliminated 11 of them to reduce the burden of the participants and the total time of completion. The final 15 items included 1 item each for timeline (acute/chronic) and timeline (cyclical), 3 items for personal control, 2 items for treatment control, 4 items for emotional representations, and 4 items for the consequences. A Greek version of the IPQ-R adapted for breast cancer is available at the official IPQ site (http://ipq.h.uib.no). However, we adapted the translation to better reflect the aims of the study as it is suggested by the developers of the scale. Views about illness are rated in a five-point Likert scale from strongly disagree (coded 1) to strongly agree (coded 5). We reversed the coding of items appropriately as instructed by the developers.

### 2.5. Emotional Impact of the Epidemic

The emotional impact of the epidemic was assessed using the four items from the IPQ-R related to emotional representations of the illness (see Section 2.4). These four items were reworded to reflect the epidemic as follows. (a) The current situation with the epidemic makes me feel angry. (b) I get depressed when I think about the epidemic. (c) When I think about the epidemic I get upset. (d) This epidemic makes me feel afraid. We combined these four items to derive a total score for the emotional impact of the epidemic (range from 4 to 20). In addition, in the analyses, we also present a binary variable corresponding to responses coded “agree or strongly agree” (i.e., coded at least 4 in each individual item) on average.

### 2.6. Other Variables

We used direct questions in order to assess all remaining sociodemographic variables. Use of alcohol was also self-reported regarding frequency, quantity on a typical day, and binge drinking. We also asked two COVID-19-related questions, the first concerning the amount of time spent out of home for reasons other than work and the second regarding exposure to media for COVID-19-related news in terms of time spent on a typical day.

### 2.7. Statistical Analysis

We used Stata version 12.0 for all analyses (StataCorp, College Station, Texas). Factor analyses of the two instruments (Brief COPE and IPQ-R) were performed with the command “factor” in Stata. Odd ratios for depression and anxiety and their 95% confidence intervals were calculated with a series of adjusted logistic regression models. All evaluations of statistical significance are based on two-sided tests using the 5% level of significance.

## 3. Results

### 3.1. Description of the Sample

Three thousand three hundred and seventy-nine (3379) adults took part in the study. The sample was predominantly female (73% of the final sample), mean age was 42 years old (SD: 12.6), 52% were married, 69.7% were employed, and 78% were living in a major city. The sample was well educated with 37% having an upper secondary or postsecondary education, 30% a university degree, and 30% a postgraduate degree. (Table A1 in the supplementary appendix gives full details of the characteristics of the participants).

Almost 25% of the sample reported financial difficulties of their household. Regarding exposure to media related to COVID-19 news, 17.37% of the sample reported a high/excessive use of several media with men slightly more likely to report such use compared to women (19.6% vs. 16.52%, *p* = 0.034). Time spent outside home, excluding time related to work, was small and only 10.36% of the sample reported being out of home for a high or excessive amount of time (15.29% of men vs. 8.5% of women, *p* < 0.001).

### 3.2. Level of Current General and Mental Health

Levels of general and mental health are shown in Table 1. It can be seen that there were high levels of mild depressive (at a cut-off of 10 or more for PHQ-9) and anxiety symptoms (at a cut-off of 3 or more on GAD-2), although these were considerably lower at a moderate level especially for depressive symptoms (PHQ‐9 ≥ 15). Fair or poor general health was reported by 11.66% of the population. All health status variables were statistically significantly higher in women except for moderate depressive symptoms which were marginally nonsignificant.

### 3.3. Factor Analysis of the Illness Perception Questionnaire

A factor analysis of the 15 items of the IPQ-R confirmed the hypothesized dimensions, namely, the “personal control” dimension (related to beliefs about the ability of taking measures to personally control the illness and self-efficacy), the “treatment control” dimension (beliefs about treatment expectancies), and the “emotional representation” dimension of the epidemic (these represent the emotional impact of the epidemic). The two items related to the timeline of the illness (acute/chronic and cyclical course) were more related to the “treatment control” dimension. Due to their nature, the items related to the consequences dimension were treated individually. (Details of the factor analysis are given in the supplementary appendix, Table A2.)

### 3.4. Emotional Impact of the Epidemic

**Figure 1:**
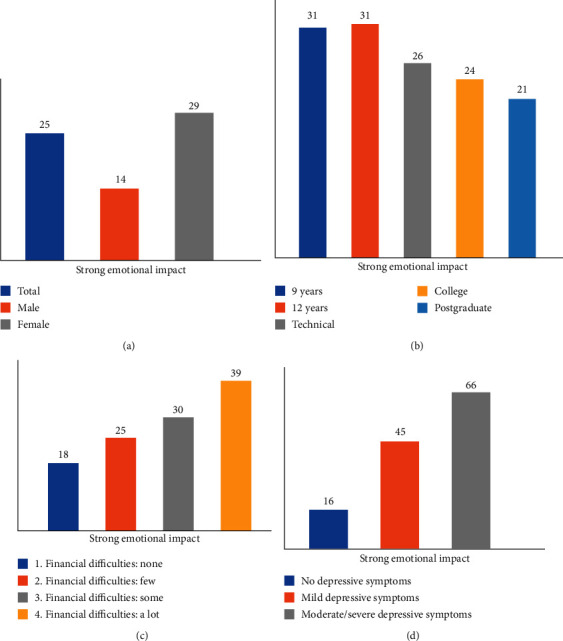
Associations of “strong emotional impact” of the COVID-19 epidemic with (a) gender, (b) educational status, (c) presence of financial difficulties, and (d) depressive symptoms in Greece (*N* = 3379, all *p* < 0.001).

The emotional impact of the epidemic was assessed with the corresponding items of the IPQ-R (see Methods). Approximately 25% of the sample showed a strong emotional impact to the epidemic (Table 2). This impact was larger in women (29% versus 14% in men), in those with a lower education (31% versus 21% in those with a postgraduate degree) and in those with a lot of financial difficulties in their household (39% versus 18% in those without difficulties). All these differences were statistically significant (*p* < 0.001, see Figure 1).

There was a strong association with levels of depressive symptoms and emotional impact to the epidemic. Approximately 45% and 66% of those with mild or moderate depressive symptoms, respectively, showed a strong emotional impact to the epidemic (compared to 16% of those with low levels of depressive symptoms, *p* < 0.001). Similar figures were reported for anxiety symptoms. A similar association with a stronger emotional impact was noted for levels of general health, with 52% of those who reported fair/poor general health showing a stronger emotional impact compared to 21% of those with better health (odds ratio: 3.84, *p* < 0.001 adjusted for age and sex).

### 3.5. Other Illness Perceptions

The sample showed high levels of personal control and self-efficacy (66.32%) as assessed with the IPQ-R (Table 2). In contrast, only 25.24% of the population had an optimistic view about potential treatments of the illness, while 27.79% viewed the illness as potentially severe (Table 2). Sense of personal control increased with age as was severity of illness (16.59% in the young versus 41.64% in the elderly). A higher educational status was also associated with higher personal control and lower levels of beliefs about the severity of illness.

Beliefs about future severe financial consequences were also very prevalent (Table 3). Stigma related to the illness was more prevalent in men compared to women and decreased with a higher educational status.

### 3.6. Use of Coping Strategies

**Figure 2:**
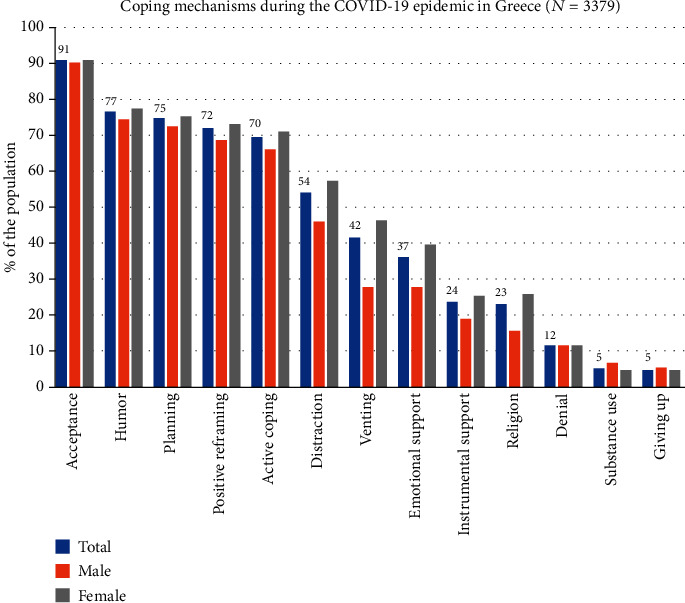
Coping strategies used during the COVID-19 epidemic in Greece (*N* = 3379). Positive/active strategies include acceptance, humor, planning, positive reframing, and active coping. Supportive/distractive strategies include distraction, venting, emotional support, and instrumental support.

The percentage of the sample that used each one of the 13 coping strategies for at least a medium amount of time is shown in Figure 2. Acceptance, humor, and planning were the three more common strategies used in both genders. (Table A3 in the supplementary appendix also presents the results in more detail.)

Using factor analytical techniques, we derived two broad factors, one positive/active comprised of 5 coping strategies and one supportive/distractive comprised of 4 coping strategies. The remaining 4 strategies were treated individually as they did not load clearly to one of the two factors (details of the factor analysis are given in the supplementary appendix, Tables A4 and A5). Positive/active strategies were more often used in the study population compared to more supportive strategies. More dysfunctional strategies, such as denial, substance use, and giving up, were more rarely used. Supportive strategies and religious coping were more likely to be used by women, while substance use by men. Denial and giving up did not differ between genders (see Table A3 in the supplementary appendix).

**Figure 3:**
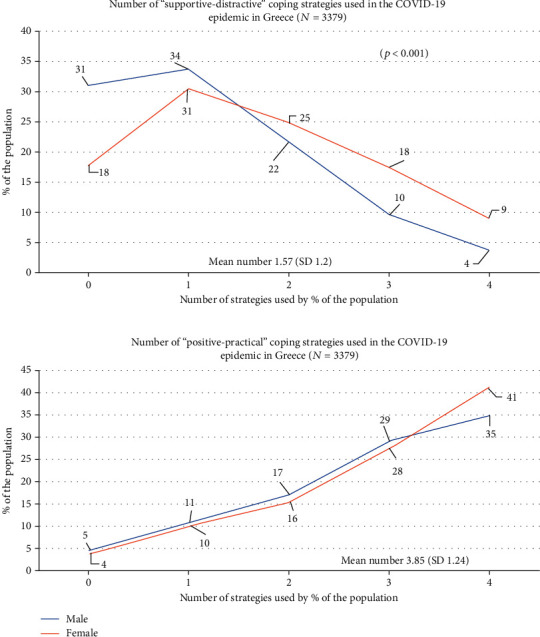
Number of coping strategies used in the COVID-19 epidemic in Greece (*n* = 3379).

Participants typically used several coping strategies at the same time: overall, they reported the use of a mean number of 3.85 positive/active strategies (SD: 1.24) versus 1.57 (SD: 1.2) supportive/distractive strategies (see Figure 3).

Regarding age and educational status, use of “planning” and religious coping increased with age. Instrumental support (getting advice from others) decreased with age. It is worth noting that two of the dysfunctional coping strategies, denial and giving up, showed a U-type association with increased use at the two age extremes (the young and the elderly). (Figure A1 in the supplementary appendix presents these results in more detail.)

Positive coping strategies were more likely to be used in those with high levels of personal and treatment control (odds ratio: 1.91 and 1.53, respectively, *p* < 0.001 adjusted for age and sex), while supportive coping strategies in those with a stronger emotional impact of the epidemic (odds ratio: 2.45, *p* < 0.001 adjusted for age and sex). Denial was less common in those with high levels of personal control (odds ratio: 0.43, *p* < 0.001 adjusted for age and sex), while giving up was less common in those with high levels of personal and treatment control (odds ratio: 0.50 and 0.51, respectively, *p* < 0.001 adjusted for age and sex).

### 3.7. Regression Analysis of Depressive Symptoms

Table 3 presents the results of the logistic regression analysis for the association of depressive symptoms (binary variable) with coping strategies adjusted for illness perceptions and other COVID-19-related variables. It can be seen that a higher score on the positive coping strategy dimension was associated with a lower prevalence of depressive symptoms, while more supportive/distractive strategies were associated with an increased prevalence. The number of positive strategies used was also negatively associated with depressive symptomatology independently. Illness perceptions related to high personal control or treatment control were also associated with fewer depressive symptoms. A stronger emotional impact of the epidemic was associated with an increased prevalence of depressive symptoms. It is also worth noting that a positive association with depressive symptoms was also found for a higher exposure to media for COVID-19-related information and more time spent outside home for activities not related to work. Finally, individuals in isolation due to potential symptoms and students showed more depressive symptoms. Similar analyses were obtained for anxiety scores (data on file).

## 4. Discussion

### 4.1. Main Findings

One in four of the participants in this survey experienced a strong emotional impact due to the COVID-19 epidemic. This effect was greater in women, and it was also associated with increasing depressive (and anxiety) symptoms and a worse level of general health. The presence of financial difficulties in the household was associated with a stronger emotional impact due to the epidemic. Despite this strong emotional impact, most participants used positive/active strategies to cope with the stress of the epidemic and this was more likely in those who showed high levels of personal control over the epidemic. In contrast, participants with a stronger emotional impact turned to more supportive coping strategies. In the multivariate analysis, increasing levels of current depressive symptoms were seen in the younger, in students, in those with a stronger emotional impact to the epidemic, in those isolated due to COVID-19-related symptoms, and those overexposed to media for COVID-19-related news. Lower levels of depression were seen in those using positive coping strategies and showing high levels of personal and treatment control.

### 4.2. Prevalence of Depressive and Anxiety Symptoms

The levels of depressive symptoms were higher compared to past periods (that preceded the 2009 Greek financial crisis) but were comparable to the levels reported during the years of financial crisis especially using the cut-off for moderate symptoms [38, 39]. A study in China [40] and another in Spain [41] also did not report higher than expected current depressive symptoms, while a study in Italy [42] reported higher levels. It is difficult to compare these levels due to the different methodologies used in these studies. Anxiety symptoms were higher compared to depression, a finding also confirmed in other surveys in China during this period [40, 43, 44]. Compared to another web survey conducted in Greece during this period [45], our study has shown increased depressive and anxiety levels probably due to the period of data collection which in our study was early April while in the Papandreou et al. study was early May [45].

### 4.3. Coping Behaviors

Most of the participants adapted to the epidemic using positive/active coping strategies. Acceptance of the epidemic was very high and denial very small. In addition, participants used several positive coping behaviors at the same time, therefore increasing their ability to adapt. At the same time, this strategy was associated with a better mental health and fewer depressive and anxiety symptoms. It is certainly possible that healthier individuals before the epidemic were more likely to adapt using more functional strategies, but given the strength of the association and the dose-response effect on depression (since the number of positive strategies used was also negatively associated with depression), it is equally likely that these behavioral strategies may have helped in mitigating the effects of the pandemic in the mental health status of the population. More emotionally focused (supportive) strategies were used mainly by participants who showed a strong emotional impact due to the epidemic. Although this was expected, this behavioral pattern was also associated with a worsening of mental health. Chew et al. [46] in their review of coping strategies during previous infectious disease outbreaks have reported similar results for the effect of positive/active strategies versus supportive strategies in the mental health status of the population.

### 4.4. Associations of Depression

Although depressive symptoms (using the typical PHQ-9 cut-off at 10) were more likely in women in the univariate analysis, this was not confirmed in the multivariate analysis. This was mainly due to the inclusion of the emotional impact variable which was the strongest correlate of depression in the analysis and was also stronger in women. Students were more likely to report depression independently of age. Other studies from Spain and Greece also reported a similar finding [41, 47]. It is likely that the extra effort required for distance education and the uncertainty regarding the progress of their studies may have played a role in this finding.

Specific illness perceptions were also associated with depressive symptoms. A higher sense of personal control over the epidemic and higher confidence in the efficacy of potential treatments were associated with fewer depressive symptoms. In contrast, a higher belief in the severity of illness was associated with a higher depressive symptomatology. Positive coping behaviors were negatively associated with depression independently of the illness perceptions.

Overexposure to media for COVID-19-related news was also associated with a higher depressive symptomatology. This is a finding that has been reported by other studies in China [43, 44, 48]. Although we cannot exclude reverse causality, it is equally likely that the way media have presented the more severe extreme of the illness may have a negative impact in mental health, especially for those overexposed to the news. On the other hand, time spent out of home was positively associated with depression, a finding that more likely reflects an inability of participants with depression to follow the restrictive rules of the lockdown.

Subjects on isolation due to symptoms were also more likely to report depression. This is a finding confirmed elsewhere as well [40] and highlights the need of mental health assessment in those in quarantine or isolation as has been suggested in a recent review [49].

### 4.5. Limitations

The convenient sample recruited through the social media is not representative of the general population, and we cannot exclude the possibility of selection bias. In addition, the cross-sectional nature of the study does not allow the investigation of the temporal association between variables. For this reason, all reported associations could be bidirectional. Although we have used well-known and validated instruments to assess the variables of interest, some of these measures have been adapted especially for the purposes of the current study and their psychometric properties have not been studied in detail. Therefore, issues of measurement bias are largely unknown and may have influenced our findings. Depressive symptoms were assessed with a well-validated instrument, but its reliability in different age groups is not known and results might differ if age-specific instruments had been used [50].

## 5. Conclusion

Our findings show that people in Greece adapted to the stress caused by the epidemic using predominantly positive/active strategies. This behavioral pattern may have resulted in the mitigation of the potential mental health effects of the epidemic. Dysfunctional strategies were rarely adopted. One key finding relevant from a public health perspective is the need to increase the sense of personal control over the epidemic and enhance self-efficacy of the population. Public health strategies often try to increase perceptions related to the vulnerability or the severity of the illness. Our findings however show that efforts to increase the perception of personal control and to disseminate practical approaches towards the reduction of risk may be more effective in protecting the mental health of the population and increasing the compliance to imposed restrictions. It is likely that part of the success of Greece to contain the spread of the outbreak can be attributed to the common sense illness perceptions and the functional behavioral patterns to cope with stress which were prevalent during this period. Finally, the role of media during the pandemic should be reexamined and specific guidance regarding the extent of reporting the extreme spectrum of the disease should be developed by health authorities and organizations.

## Figures and Tables

**Table 1:** Level of general and mental health of the sample during the COVID-19 epidemic in Greece (*N* = 3379).

	Male	Female	Total sample	*p*
*(A) Mental health*	*Continuous responses: mean (SD)*			
Depressive symptoms (scores on the PHQ-9) Anxiety symptoms (scores on the GAD-2)	5.95 (4.64) 1.69 (1.40)	7.25 (4.70) 2.04 (1.40)	6.90 (4.72) 1.95 (1.41)	**<0.001** **<0.001**
	*Binary responses: % of the sample*			
PHQ‐9 ≥ 10 (mild depressive symptoms)	18.11%	24.87%	23.02%	**<0.001**
PHQ‐9 ≥ 15 (moderate depressive symptoms)	6.51%	8.55%	7.99%	0.051
GAD‐2 ≥ 3 (mild anxiety)	21.04%	30.24%	27.73%	**<0.001**
GAD‐2 ≥ 4 (moderate anxiety)	11.28%	15.34%	14.23%	**0.003**
*(B) General health*				
Fair or poor level of health (self-reported)	8.57%	12.82%	11.66%	**0.001**

**Table 2:** Intensity of perceptions (agree/strongly agree) about the COVID-19 epidemic and its consequences in Greece (*N* = 3379).

Dimension	Male	Female	*Total sample*	*p*
High personal control	65.94%	66.46%	66.32*%*	0.776
High treatment control	26.25%	24.87%	25.24*%*	0.411
Strong emotional impact	13.88%	28.65%	24.62*%*	**<0.001**
Longer epidemic duration	47.40%	48.19%	*47.97%*	0.682
High severity of illness	26.03%	28.45%	27.79*%*	0.162
Severe financial consequences	69.52%	73.83%	72.65*%*	**0.012**
Stigma related to the illness	28.85%	25.40%	26.34%	**0.042**

**Table 3:** Association of depressive symptoms (PHQ-9 scores ≥ 10) with coping strategies, illness perceptions, and other variables during the COVID-19 epidemic in Greece (*N* = 3379).

Variable	Adjusted OR^1^	95% CI^2^	*p* value
Gender			
Men	1.00	Ref	
Women	1.12	0.88–1.41	0.35
Age	0.98	0.96–0.99	<0.001
Married (compared to singles)	0.69	0.52–0.93	0.01
Being a student	1.72	1.16–2.54	0.006
Financial difficulties			
No	1.00	Ref	
Yes	2.05	1.66–2.53	<0.001
Alcohol consumption			
Abstinent/small frequency	1.00	Ref	
Moderate frequency	1.02	0.83–1.25	0.846
High frequency	1.79	1.26–2.53	0.001
Coping strategies			
Coping: positive/active (scores)	0.88	0.81–0.95	0.001
Coping: supportive/distractive (scores)	1.15	1.05–1.27	0.004
Number of positive coping strategies used	0.80	0.69–0.94	0.005
Illness perceptions			
Illness beliefs: high personal control			
No	1.00	Ref	
Yes	0.79	0.65–0.96	0.02
Illness perceptions: high treatment control			
No	1.00	Ref	
Yes	0.62	0.49–0.79	<0.001
Illness perceptions: strong emotional representations			
No	1.00	Ref	
Yes	4.09	3.31–5.04	<0.001
Illness perceptions: high severity of illness			
No	1.00	Ref	
Yes	2.08	1.66–2.60	<0.001
COVID-19 related			
In isolation due to symptoms			
No	1.00	Ref	
Yes	2.79	1.42–5.49	0.003
Exposure to media for COVID-19-related news			
Low–typical	1.00	Ref	
High–excessive	1.76	1.38–2.24	<0.001
Time out of home (not work related)			
Low–typical	1.00	Ref	
High–excessive	2.68	1.18–6.10	0.02

^1^OR: odds ratios adjusted for all other variables of the table plus educational status, employment status, locality, number of children, number of persons living at home, number of supportive coping strategies used, being a person with a susceptible illness, being a carer of a susceptible person, having a business that stayed open during the epidemic, and being a health professional. All variables omitted were not statistically significantly associated with depressive symptoms; ^2^CI: confidence interval.

## Data Availability

Data of this study are available from the authors upon request. Please request data from the first author Petros Skapinakis (p.skapinakis@gmail.com).

## Supplementary Materials

Supplementary Materials This appendix has been supplied by the authors to give readers additional information about the research reported in this paper. Contents of the Appendix. Section 1: Table A1: basic description of the sample. Section 2: Table A2: factor analysis of IPQ-R items. Section 3: Table A3: use of coping strategies by gender. Section 4: factor analysis of Brief COPE items. Table A4: initial factor analysis. Table A5: second factor analysis. Section 5: Figure A1: selected coping mechanisms by age and educational status.
