# Supplementary material for: Depression and Its Relationship with Coping Strategies and Illness Perceptions during the COVID-19 Lockdown in Greece: A Cross-Sectional Survey of the Population
Source: Depress Res Treat. 2020 Aug 26;2020:3158954. doi: 10.1155/2020/3158954 (PMC7450302; doi:10.1155/2020/3158954)
Supplement: Supplementary Materials — This appendix has been supplied by the authors to give readers additional information about the research reported in this paper. Contents of the Appendix. Section 1: Table A1: basic description of the sample. Section 2: Table A2: factor analysis of IPQ-R items. Section 3: Table A3: use of coping strategies by gender. Section 4: factor analysis of Brief COPE items. Table A4: initial factor analysis. Table A5: second factor analysis. Section 5: Figure A1: selected coping mechanisms by age and educational status. [file 3158954.f1.docx]

**Supplementary Appendix**

This appendix has been supplied by the authors to give readers additional information about the research reported in this paper

**Supplement to: Skapinakis et al.** **Depression and its relationship with coping strategies and illness perceptions during the COVID-19 lockdown in Greece: A cross-sectional survey of the population**

**Contents of the Appendix**

**Section 1. Table A1.** Basic Description of the Sample

**Section 2. Table A2.** Factor analysis of IPQ-R Items

**Section 3. Table A3.** Use of Coping Strategies by gender

**Section 4. Factor analysis of Brief Cope Items**

**Tables A4.** Initial factor analysis

**Table A5.** Second factor analysis

**Section 5: Figure A1.** Selected coping mechanisms by age and educational status

**Appendix - Section 1**

**Table A1. Basic Description of the Sample (N=3379).**

|  | N | % |
| --- | --- | --- |
| **Gender**  Men  Women | 922  2457 | 27.29 %  72.71 % |
| **Age**  <=29  30-39  40-49  50-59  60+ | 663  703  1033  687  293 | 19.62 %  20.80 %  30.57 %  20.33 %  8.67 % |
| **Marital Status**  Single  Married  Divorced / Separated  Widowed / other | 1180  1762  322  115 | 34.92 %  52.15 %  9.53 %  3.40 % |
| **Educational Qualifications**  Up to Lower Secondary  Upper Secondary Education  Post-secondary / Technical Vocational  University Degree  Postgraduate | 55  473  789  1021  1041 | 1.63 %  14.00 %  23.35 %  30.22 %  30.81 % |
| **Employment Status**  Full-time / part-time  Looking after house  Unemployed  Retired  Other Economically Inactive | 2354  109  225  198  493 | 69.67 %  3.23 %  6.66 %  5.86 %  14.59 % |
| **Type of locality**  Athens - Thessaloniki  Large Cities (>50000)  Smaller Cities (<50000)  Rural | 1569  1070  307  433 | 46.43 %  31.67 %  9.09 %  12.81 |
| **Presence of financial difficulties**  None  Few  Some  A lot | 961  1565  658  195 | 28.44 %  46.32 %  19.47 %  5.77 % |
| **Level of general health**  Excellent  Very Good  Good  Fair  Poor | 516  1477  992  353  41 | 15.27 %  43.71 %  29.36 %  10.45 %  1.21% |
| **Exposure to media for Covid-19 related news**  Low - Typical  High - Excessive | 2792  587 | 82.63 %  17.37 % |
| **Time out of home (not work-related)**  Low - Typical  High - Excessive | 3029  350 | 89.64 %  10.36 % |

**Appendix - Section 2. Factor analysis of IPQ-R Items**

**Table A2.** Factor analysis of the IPQ-R items

| IPQ-R item | Factor 1 | Factor 2 | Factor 3 |
| --- | --- | --- | --- |
| ipq1^1^ | -0.0536 | 0.7074 | -0.1437 |
| ipq2^5^ | 0.1134 | -0.3130 | 0.4524 |
| ipq3^4^ | -0.2083 | 0.3217 | -0.4472 |
| ipq4^1^ | 0.0225 | -0.6943 | -0.1568 |
| ipq5^4^ | 0.2397 | 0.1657 | 0.1210 |
| ipq6^2^ | 0.0674 | -0.1002 | -0.7008 |
| ipq7^3^ | 0.5744 | 0.3068 | -0.1379 |
| ipq8^4^ | 0.4000 | 0.1835 | 0.0372 |
| ipq9^3^ | 0.8189 | 0.0289 | 0.0252 |
| ipq10^6^ | -0.1751 | 0.0230 | -0.4834 |
| ipq11^3^ | 0.8334 | 0.0143 | 0.0406 |
| ipq12^4^ | 0.4785 | -0.0623 | 0.4807 |
| ipq13^2^ | 0.1752 | 0.2879 | 0.6154 |
| ipq14^1^ | 0.1211 | 0.7820 | 0.1031 |
| ipq15^3^ | 0.7507 | -0.0771 | 0.2258 |

1: items related to Personal Control

2: items related to Treatment Control

3: Items related to Emotional Representation

4: Items related to Consequences

5: Item related to Cyclical Timeline

6: Item related to Acute/Chronic Timeline

**Appendix - Section 3**

**Table A3: Use of Coping Strategies (at least a medium amount of time) in the Covid-19 epidemic in Greece (N=3379)**

| **Latent Factor^1^** | **Coping Strategy** | ***Total*** | **Male** | **Female** | **p^2^** |
| --- | --- | --- | --- | --- | --- |
| Positive/Active | Acceptance | *91.03%* | 90.35 | 91.29 | 0.393 |
| Positive/Active | Humor | *76.86%* | 74.84 | 77.61 | 0.088 |
| Positive/Active | Planning | *74.93%* | 72.56 | 75.82 | 0.051 |
| Positive/Active | Positive Reframing | *72.18%* | 69.09 | 73.34 | **0.014** |
| Positive/Active | Active Coping | *69.90%* | 66.16 | 71.31 | **0.004** |
| Supportive / Distractive | Distraction | *54.28%* | 46.20 | 57.31 | **0.001** |
| Supportive / Distractive | Venting | *41.73%* | 28.20 | 46.81 | **0.001** |
| Supportive / Distractive | Emotional Support | *36.67%* | 27.98 | 39.93 | **0.001** |
| Supportive / Distractive | Instrumental Support | *23.94%* | 19.20 | 25.72 | **0.001** |
| NA^3^ | Religion | *23.23%* | 15.94 | 25.97 | **0.001** |
| NA | Denial | *11.93%* | 11.82 | 11.97 | 0.909 |
| NA | Substance Use | *5.47%* | 6.83 | 4.97 | **0.034** |
| NA | Giving up | *5.30%* | 5.53 | 5.21 | 0.710 |

**^1^** According to the factor analysis (see section 3.3); **^2^** p-value for the gender difference; **^3^** Not applicable, items excluded from the factor analysis (see section 3.3)

**Appendix - Section 4. Factor analysis of Brief Cope Items**

An exploratory factor analysis of the thirteen selected brief cope items identified two potential latent factors with eigenvalues more than one (Table A2 in this supplementary appendix). Items that were not clearly loaded to one of the two factors were excluded (these were the items related to: denial, use of alcohol or other substances, giving up and religious coping). We then repeated the factor analysis with the remaining 9 items retaining two factors (Table A3). According to this analysis the first factor comprised of 5 items (Active Coping, Positive Reframing, Planning, Humor and Acceptance) and the second factor of 4 items (Distraction, Emotional Support, Instrumental Support and Venting). Items were at least moderately loaded (>0.40) to their corresponding factor (with the exception of Distraction that loaded at 0.30 – see Table A1). The first factor could be considered as the positive/active factor and the second as the supportive/distractive factor. For the regression analyses we summed the two factors to derive two continuous variables (with a range from 5 to 20 for the positive and 4 to 16 for the supportive) and two binary variables (with cut-offs at 16 and 12 respectively, corresponding to a mean value of at least a “medium amount of time” of using the corresponding coping strategy. The excluded four coping strategies were treated as individual items in the analyses.

**Table A4. Initial factor analysis (items in bold excluded from the next factor analysis)**

| Coping Strategy | Factor 1 | Factor 2 | Factor 3 | Factor 4 | Factor 6 | Factor 7 |
| --- | --- | --- | --- | --- | --- | --- |
| nbc1 | 0.1908 | 0.2596 | 0.0975 | 0.2691 | 0.0742 | -0.0030 |
| nbc2 | 0.4050 | 0.2498 | -0.0911 | 0.2960 | -0.0836 | 0.0024 |
| **nbc3** | -0.0896 | 0.0506 | 0.5526 | 0.0289 | 0.0162 | 0.0019 |
| **nbc4** | -0.0929 | 0.1389 | 0.1479 | -0.0019 | 0.2503 | 0.0012 |
| nbc5 | 0.0550 | 0.6595 | 0.0121 | 0.0239 | 0.0310 | -0.0016 |
| nbc6 | 0.0379 | 0.5994 | 0.0194 | 0.0557 | -0.0296 | 0.0014 |
| **nbc7** | -0.2600 | 0.0729 | 0.3454 | -0.0694 | 0.1729 | 0.0066 |
| nbc8 | -0.0348 | 0.4664 | 0.0651 | -0.0006 | 0.0065 | 0.0065 |
| nbc9 | 0.5928 | -0.0181 | -0.1700 | -0.0008 | -0.0352 | -0.0017 |
| nbc10 | 0.5000 | 0.1116 | -0.1232 | 0.1379 | -0.0809 | 0.0069 |
| nbc11 | 0.4778 | 0.0312 | -0.1125 | -0.0430 | 0.1181 | -0.0054 |
| nbc12 | 0.2939 | 0.0056 | -0.5126 | 0.0309 | 0.0199 | 0.0031 |
| **nbc13** | 0.1674 | 0.1642 | 0.1091 | 0.0569 | -0.1800 | 0.0107 |

| Factor | Eigenvalues |
| --- | --- |
| Factor1 | 1.70 |
| Factor2 | 1.25 |
| Factor3 | 0.39 |

**Table A5. Second factor analysis with 9 items**

| Coping Strategy | Factor 1 | Factor 2 |
| --- | --- | --- |
| nbc1 (Distraction) | 0.1908 | **0.3086** |
| nbc2 (Active Coping) | **0.4471** | 0.2825 |
| nbc5 (Emotional Support) | 0.0503 | **0.6546** |
| nbc6 (Instrumental Support) | 0.0416 | **0.5989** |
| nbc8 (Venting) | -0.0490 | **0.4586** |
| nbc9 (Positive Reframing) | **0.5973** | -0.0315 |
| nbc10 (Planning) | **0.5351** | 0.1162 |
| nbc11 (Humor) | **0.4818** | 0.0145 |
| nbc12 (Acceptance) | **0.4167** | -0.0334 |

**Appendix - Figures**

**Figure Α1**
